# Effect of *Cymbopogon martinii, Foeniculum vulgare*, and *Trachyspermum ammi* Essential Oils on the Growth and Mycotoxins Production by *Aspergillus* Species

**DOI:** 10.1155/2014/874135

**Published:** 2014-06-23

**Authors:** Negero Gemeda, Yimtubezinash Woldeamanuel, Daniel Asrat, Asfaw Debella

**Affiliations:** ^1^Traditional & Modern Medicine Research Directorate, Ethiopian Health & Nutrition Research Institute, P.O. Box 1242, Addis Ababa, Ethiopia; ^2^Department of Microbiology, Immunology & Parasitology, Addis Ababa University, P.O. Box 9086, Addis Ababa, Ethiopia

## Abstract

This study was performed to investigate effect of essential oils on *Aspergillus* spore germination, growth, and mycotoxin production. *In vitro* antifungal and antiaflatoxigenic activities of *Cymbopogon martinii, Foeniculum vulgare,* and *Trachyspermum ammi* essential oils were carried out on toxigenic strains of *Aspergillus* species. Plant materials were hydrodistilled for 4-5 h in Clevenger apparatus. 0.25 *μ*L/mL, 0.5 *μ*L/mL, 1 *μ*L/mL, 2 *μ*L/mL, and 4 *μ*L/mL concentrations of each essential oil were prepared in 0.1% Tween 80 (V/V). *T. ammi* oil showed highest antifungal activity. Absolute mycelial inhibition was recorded at 1 *μ*L/mL by essential oils of *T. ammi*. The oil also showed complete inhibition of spore germination at a concentration of 2 *μ*L/mL. In addition, *T. ammi* oil showed significant antiaflatoxigenic potency by totally inhibiting toxin production from *A. niger* and *A. flavus* at 0.5 and 0.75 *μ*L/mL, respectively. *C. martinii, F. vulgare,* and *T. ammi* oils as antifungals were found superior over synthetic preservative. Moreover, a concentration of 5336.297 *μ*L/kg body weight was recorded for LC50 on mice indicating the low mammalian toxicity. In conclusion, the essential oils from *T. ammi* can be a potential source of safe natural food preservative for food commodities contamination by *Aspergillus* species.

## 1. Introduction

Food borne diseases caused by fungal pathogens are still the major public health problems in this era of advancement in food production technologies [1]. Quarter of the world's food commodities have been wasted due to the contamination by toxic fungi or by fungal metabolic products [2]. Improper storage condition offers favorable environment for the growth of* Aspergillus *species [2] and production of mycotoxins [2]. Consumptions of such contaminated food lead to a serious cases of illness and mycotoxicoses [3, 4]. Among these, aflatoxicosis can manifest both acute (hemorrhage, acute liver damage, edema, and death) and chronic toxicological effects of cancer, mutagenicity, immune suppression, birth defects, estrogenic, gastrointestinal, urogenital, vascular, kidney and nervous system disorder [1, 2, 5]. In Africa, particularly in parts of sub-Saharan Africa, about 250,000-hepatocarcinoma related deaths occur annually due to aflatoxin ingestion alone [1, 4].

Current consumer desires safe and uncontaminated food commodities throughout the supply chain (from “farm to plate”) [2]. Several synthetic preservatives have been effectively used in management of food contamination by* Aspergillus *species but their continuous application has led to the development of fungal resistance [6], number of environmental and health problems [7–9]; hormonal imbalance and spermatotoxicity [10]; and also some individuals produce allergic reactions to these substances [11]. However, natural products could potentially serve as effective alternatives of synthetic chemicals for the control of food contamination by* Aspergillus* species [12, 13]. Among natural products, essential oils (EOs) of aromatic plants are gaining interest as food additives and widely accepted by consumers because of their relatively low toxicity, highly volatile, transient, and biodegradability nature [14, 15]. European Union allowed the use of essential oils in food and aromatherapy [16]. So, EOs with antimicrobial activity are possible candidates for the preservativation of food commodities against* Aspergillus *species [17].

*Cymbopogon martinii *L.*, Foeniculum vulgare* Miller, and* Trachyspermum ammi* L. Sprague ex Turrillare medicinal aromatic plants of Ethiopia. They are used traditionally as food additives and also are used for the treatment of various diseases [18]. However, as far as our knowledge is concerned, there is no reliable evidence that indicated that these plants essential oils have fungitoxic and antiaflatoxigenic potentials against aflatoxigenic* Aspergillus *species in Ethiopia. The aim of this study was to evaluate the effect of essential oils on growth, spore, and mycotoxin production of* Aspergillus *species that could alternate synthetic chemical preservatives.

## 2. Material and Method

### 2.1. Chemicals and Media

Our media, chemicals, and solvents used in the study were obtained from different companies in different countries. Potato Dextrose Agar was obtained from HiMedia Laboratories Pvt. Ltd., India; Sabouraud dextrose agar from Oxoid Ltd., England; Sabouraud dextrose broth from Difco, Ltd., England; sucrose and yeast extract from Labort fine Chem Pvt. Ltd., India; Aflatoxin Mix Kit-M from Supelco, USA; anhydrous Sodium sulphate, MgSO_4_·7H_2_O, Potassium hydroxide, Vanillin, and Silicagel60 thin layer chromatography plate 0.2 mm from Merck-Schuchardt, Germany; potasium nitrate from Rhone Poulenc, US; sodium benzoate from Codex Farmacopia, Italy; thymol, Chloroform, Ethyl acetate, sulphuric acid, Tween 20, and Tween 80 from Sigma-Aldrich Chemie, Germany; acetone from Labort Fine Chem Pvt. Ltd., India; ethanol absolute and Methanol from Finken, India; and toluene from AnalaR, England.

### 2.2. Plant Materials

*Cymbopogon martinii* (aerial part) was collected from the botanical garden of TMMRD, EHNRI, Ethiopia;* Foeniculum vulgare *(leaf lamina and leaf sheath) from Shashamane, Ethiopia; and* Trachyspermum ammi *(fruits) from Tepi, Ethiopia. Following collection, identification of plants was carried out in Traditional and Modern Drug Research Department of the Ethiopian Health and Nutrition Research Institute (EHNRI). Fresh areal parts of* C. martinii, F. vulgare*; and dried fruits of* T. ammi *(250 g) were placed in a 5 L round-bottom distillation flask and the plant material was wetted with 3 L distilled water. The essential oils were obtained by hydrodistillation using Clevenger-type apparatus for continuous 3 h. The volatile oil was taken from the upper layer. The excess aqueous layer was further portioned using dichloromethane to extract and enrich the essential oil from the water layer. The organic layer (dichloromethane extract) was filtered and dried with anhydrous sodium sulfate and concentrated using rotary evaporator to give the crude essential oil.

### 2.3. Phytochemical Analysis of the Essential Oils

#### 2.3.1. TLC Analysis

Following the extraction of essential oils that are intended for biological assay, a portion of essential oils were subjected to thin-layer chromatography (pre coated Silica gel G60F254) finger print analyses. Wagner and Bladts' procedure for plant drug analysis was used for the developing of chromatogram and for the identification of major secondary metabolites responsible for biological activity [19].

#### 2.3.2. GC-Analysis

Gas chromatographic analysis of the oil of* T. ammi *was performed on a Shimadzu GC-2010 system, with split mode. The column used was a ZB-1MS equivalent to OV-1, fused silica capillary column 30 m × 0.25 mm i.d., film thickness 0.25 *μ*m, coated with 5% diphenyl-95% polydimethylsiloxane, and operated with the following oven temperature programs: 50°C, held for 2 min, rising at 3°C/min to 210°C. Injection temperature and volume were 250°C and 1.0 *μ*L, respectively; injection mode was split; split ratio was 10 : 1; carrier gas was nitrogen at 65.2 cm/s linear velocity and inlet pressure was 100 KPa; detector temperature was 270°C; nitrogen flow rate was 52.1 mL/min; air flow rate was 400 mL/min; make-up 32, (H2/air) flow rate was 40 mL/min; and sampling rate was 40 ms.

### 2.4. Fungal Strains

Toxigenic strains of* Aspergillus flavus *(AF001, AF006′, AF009, AF019, AF027, and AF037) and* Aspergillus niger *(AN002) were selected for this study. The strains were isolated from food commodities that were collected from different local markets in Addis Ababa prior to the study. In addition, standard strains of* Aspergillus flavus *(ATCC 13697) and* Aspergillus niger *(ATCC 10535) were used in the study as a control. The standard strains were obtained from Microbiology laboratory, Traditional and Modern Medicine Research Directorate, Ethiopian Health and Nutrition Research Institute.

### 2.5. Antifungal Activity

#### 2.5.1. Determination of Sporicidal Activity

Sporicidal activities of* C. martinii, F. vulgare, *and* T. ammi* were conducted using spore germination assay according to standard reference method [20]. The test organisms were grown on PDA medium for sporulation and spores were harvested after 10 days of incubation. Spores were collected by adding 5 mL of sterile water containing 0.1% (v/v) Tween 80 to each Petri dish and rubbing the surface with a sterile L-shaped spreader (3 times). The suspension was collected and then centrifuged at room temperature at 2000 rpm for 5 min. The supernatant was discarded and recentrifuged until 1 mL of highly concentrated spore solution remained. A haemocytometer slide was used to count spore production to have approximately 10^8^ spore/mL [21].

Concentrations of essential oils were prepared in 5 mL of sabouraud dextrose broth in 100 mL flask and then 1 mL of the spore suspension was added to each flask. The flasks were then incubated for 24 h at 25°C on a rotary shaker (60 rpm) as to evenly disperse the oil throughout the broth. At the end of the incubation period, germinated spores were observed using a light microscope at 400x magnification. Experiment was performed in triplicate and the extent of spore germination was assessed by looking for the presence of germ tubes. Results were expressed in terms of the percentage of spores germinated as compared to the control from the average of the triplicates. Percentage spore germination inhibition is calculated according to the following formula:

(1) $$\begin{array}{c} \%\text{Spore}\;\text{Germination}\;\text{inhibition}=\frac{\left(\text{sc}-\text{st}\right)}{\left(\text{sc}\right)}\times 100, \end{array}$$

where sc is the average number of spore germinated in control set and st is the average number of spore germinated in test set.

#### 2.5.2. Determination of Minimum Inhibitory Concentration

The minimum inhibitory concentrations (MICs) of* C. martinii, F. vulgare, *and* T. ammi* essential oils were determined using agar dilution methods [20, 22]. Two-fold serial dilutions of essential oils in sabouraud dextrose agar were made to have a final concentration of 0.25 *μ*L/mL, 0.5 *μ*L/mL, 1 *μ*L/mL, 2 *μ*L/mL, 4 *μ*L/mL, and 8 *μ*L/mL. The experiments were performed in triplicates. Control plates, containing no essential oils, were run simultaneously. The agar surface of the plates containing the dilution of essential oils and the control plate are inoculated by five millimeter discs of the test fungi taken from advancing edge of 7-day-old cultures. The plate containing the lowest concentration of essential oils was seeded first. Control plates were seeded last to insure that viable organisms were present throughout the procedure. Incubate the inoculated plates at 26 ± 2°C for seven days before being read. End-points for each EOs are best determined by placing plates on a dark background and observing the lowest concentration that inhibits visible growth, which is recorded as the MIC. The MIC of essential oil was usually recorded in microliter per milliliter.

#### 2.5.3. Determination of Mycelial Dry Weight

The effect of essential oils on mycelial dry weight was evaluated in sabouraud dextrose broth [20]. Two-fold dilution of* T. ammi* essential oil in sabouraud dextrose broth was made by adding one part of oil into each nine part of sabouraud dextrose broth in a 100 mL flask to have a final concentration of 0.25 *μ*L/mL, 0.5 *μ*L/mL, 1 *μ*L/mL, 2 *μ*L/mL, and 4 *μ*L/mL. The experiments were performed in triplicates. The flasks were aseptically inoculated with 0.36 mL spore suspensions (*≈*10^6^ spore/mL). The flask containing the lowest concentration of EOs was seeded first. Control plates were seeded last to insure that viable organisms were present throughout the procedure. Incubate the inoculated plates at 26 ± 2°C for seven days before being read. Flasks containing mycelia were filtered through Whatman filter number 1 and then were washed with distilled water. The mycelia were placed on preweighed Petri plates and were allowed to dry at 60°C for 6 h and then at 40°C overnight. The flasks containing dry mycelia were weighed. Percentage of growth inhibition on the basis of dry weight was calculated as

(2) %mycelial  dry  weight  =control  weight−sample  weightcontrol  weight×100.

### 2.6. Efficacy of* T. ammi* Oil in Arresting Aflatoxin Elaboration

The methods that have been adopted by Kumar et al. [23] were used to determine antiaflatoxigenic efficacy of EOs with lowest MIC on* A. flavus* using SMKY broth medium (Sucrose, 200 g; MgSO_4_·7H_2_O, 0.5 g; KNO_3_, 0.3 g; yeast extract, 7.0 g; distilled water, 1000 mL; and pH, 5.6 ± 0.2). Different concentrations of the oil (0.1, 0.2, 0.3, 0.4, 0.5, 0.6, 0.7, 0.8, 0.9, and 1.0 *μ*L/mL) were prepared separately by dissolving their requisite amount in 0.5 mL 5% Tween 20 and then mixing it with 24.5 mL of SMKY medium in 100 mL Erlenmeyer flask [23]. The control sets were kept parallel to the treatment sets without essential oil. Then flasks were inoculated aseptically with 1 mL spore suspension (*≈*10^6^ spores/mL) prepared in 0.1% Tween 80 and incubated at 27 ± 2°C for 10 days. The content of each flask was filtered (Whatman filter paper number 1). The filtrate was extracted with 20 mL chloroform in a separating funnel and the extracts were passed through anhydrous sodium sulphate kept in Whatman filter paper number 42. The extracts were evaporated till dryness on water bath at 70°C. Dry residues were dissolved in 1 mL chloroform and 50 *μ*L of chloroform extract spotted on TLC plate (20 × 20 cm^2^ of Silica gel-G60F254) then developed in chloroform: acetone (9 : 1 v/v). The intensity of aflatoxin was observed in Ultraviolate Fluorescence Analysis Cabinet at an excitation wavelength of 366 nm.

### 2.7. Animal Trials to Determine Safety Limit of the Oils

The safety limit of the best fungitoxic essential oils was determined by recording LC50 value on mice following the protocol of Kumar and his collaborator [23]. Mice with an average weight and age (35 g, 3 months) were selected as test animals for the mammalian toxicity experiments. Requisite amount of essential oils was mixed properly with Tween 80 to prepare different solutions containing desired dose of essential oils. The mice were administered with 0.5 mL of each solution of essential oils orally separately through a gavage syringe to each set containing 10 mice (equal proportion for gender). In control sets equal volume of Tween 80 was given to mice. After 72 h, the mortality of the animals was recorded and LC50 was calculated in terms of per kg body weight of mice using SPSS version 20.0 and Minitab version 16 computer software's by Probit analysis.

### 2.8. Statistical Analysis

All the measurements were replicated three times for each treatment and data were entered into excel spreadsheet and are presented as mean ± SE/SD. Significant differences between strains aflatoxin producer and non-producer; between treatment and control were analyzed using statistical software (SPSS 20.0; Chicago, IL, USA) at 95% level of confidence by Chi-square analysis. Data were first tested for normality and then subjected to one-way analysis of variance (ANOVA) using statistical software (SPSS 20.0; Chicago, IL, USA and Minitab 16.0, England). Significant differences between mean values were determined using Tukey's and Dunken's multiple range tests following one-way ANOVA and *P* values <0.05 were considered as significant.

## 3. Results

**Figure 1:**
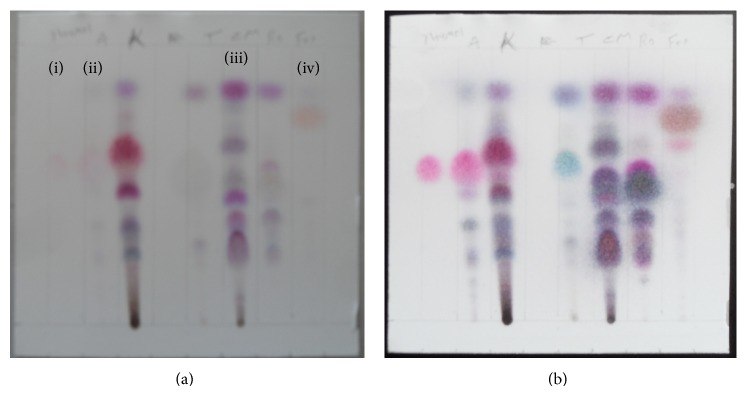
TLC fingerprint (a; b = before and after heating at 110°C for 5 minute, resp.) of thymol standard and essential oils of* Trachyspermum ammi; Cymbopogon martinii; *and* Foeniculum vulgare, *from (i) to (iv), respectively.

TLC fingerprint was used for screening of essential oils as bioactive compounds were separated in a sequence of different zones and characterized by the value of retention factors (Rf) in Toluene, ethyl acetate (9.3 : 0.7) solvent system and the color of zone they produce after being treated with detection reagent (vanillin-sulfuric acid). Each plant essential oil has shown quite different thin layer chromatography finger print (Table 1 and Figure 1). TLC screening indicated the presence of many terpenoids in the essential oil tested which was confirmed by the presence of different colored spots. The highest number of spots was obtained in the chromatogram of* C. martinii* essential oil that showed distinctive 8 spot/bands, while* F. vulgare* and* T. ammi* essential oils were separated in four different spot/bands when visually observed after treatment with vanillin-sulphuric acid reagent.

A total of 14 components were shown on our GC analysis of* T. ammi *essential oil accounting for 100% of the total amount (Table 2). Thymol (51.5%) was the major constituent of* T. ammi* essential oil followed by Carvacrol (28.5%), para-cymene (6.2%), gamma-terpinene (5.2%), and beta-pinene (5.1%) main constituents.

Each tested concentration of essential oils showed notable inhibition of* Aspergillus flavus* spore germination.* T. ammi* essential oils have pronounced spore germination inhibition effect on tested organisms. Absolute spore germination was recorded for essential oils of* T. ammi, C. martinii,* and* F. vulgare* at a concentration of 1 *μ*L/mL, 2 *μ*L/mL, and 4 *μ*L/mL, respectively. Moreover, the amount of essential oils tested has significant spore inhibition effect (*P* < 0.05) (Table 3).

The mean score for antifungal activity of essential oils were revealed by Table 4. A remarkable antifungal activity against the growth of aflatoxigenic strains of* Aspergillus flavus* was recorded after the treatment with different concentrations of* T. ammi *essential oil followed by* C. martinii *essential oil and* F. vulgare* essential oil. Increased mycelial expansions were observed at lower concentration of essential oils while absolute inhibitions of mycelial expansion were observed at higher concentration indicating dose-dependent activities. Interestingly, the minimum inhibitory concentration was recorded at 1 *μ*L/mL for* T. ammi* and 4 *μ*L/mL for both* C. mertinii* and* F. vulgare*. At these points, the plant essential oil completely inhibited the growth of all isolated fungal strains* A. flavus* (Table 4).

All the three essential oils were documented to have better fungitoxic activity against aflatoxigenic* Aspergillus *species when compared to synthetic chemical preservative sodium benzoate (Table 5). Statistical results showed that both the kind and the concentration of essential oil have significant effect (*P* < 0.05). The most promising MIC was recorded by* T. ammi* at 1 *μ*L/mL against* A. flavus* and* A. niger*.

The effect of* T. ammi* essential oil on the dry mycelial weight of* Aspergillus *species in sabouraud dextrose broth is presented in Table 6. Results of statistical analysis showed that each tested concentration of essential oils has significantly different mycelial dry weight inhibition (*P* < 0.05). It can be clearly seen that a complete inhibition of mycelial dry weight is at a concentration of 1, 2, and 4 *μ*L/mL. At least a 12, 43, and 71% of dry mycelia biomass weight suppression was recorded at 0.25, 0.5, and 0.75 *μ*L/mL, respectively, against* Aspergillus *species. A dose-dependent suppression of mycelial growth of* Aspergillus *species was observed, as the higher concentration of the* T. ammi *essential oil inhibited hundred percent of the mycelial growth.

Table 7 shows antiaflatoxigenic potentials of* T. ammi *essential oil against aflatoxigenic strains of* Aspergillus *species (*A. flavus *and* A. niger*). It has been documented from this study's chromatogram that the aflatoxin production in SMKY liquid medium was reduced by the essential oils of* T. ammi *in dose-dependent manner. Aflatoxin production was completely inhibited at a concentration of 0.50 *μ*L/mL for strains of* A. niger* and at the concentration of 0.75 *μ*L/mL* for A. flavus*. These concentrations were less than those which are recorded for minimum inhibitory concentration and absolute mycelial dry weight inhibiting concentration (1 *μ*L/mL). In all untreated control (Tween 20 5%), high level of aflatoxin production was observed in all aflatoxigenic* Aspergillus *species.

During the safety limit tests all the test animals were observed closely for up to 14 days; symptoms of toxicity, recovery, and death were noted. No sign of toxicity was recorded for the mice in groups of control, 3,000, 3,500, and 4,000 *μ*L/kg body weights. The majority of mice from groups 4,500, 5,000, and 5,500 have showed hypoactivity (decreased motility, debiting effect) and decreased feed intake. Mice from groups 6,000 and 7,000 *μ*L/kg have shown the sign of prostration, anaesthesia and muscle spasm within 30 minutes and were followed by deaths within the 24 hour. The mice that have shown hypoactivity and decreased feed intake have recovered within 24 hours.

**Figure 2:**
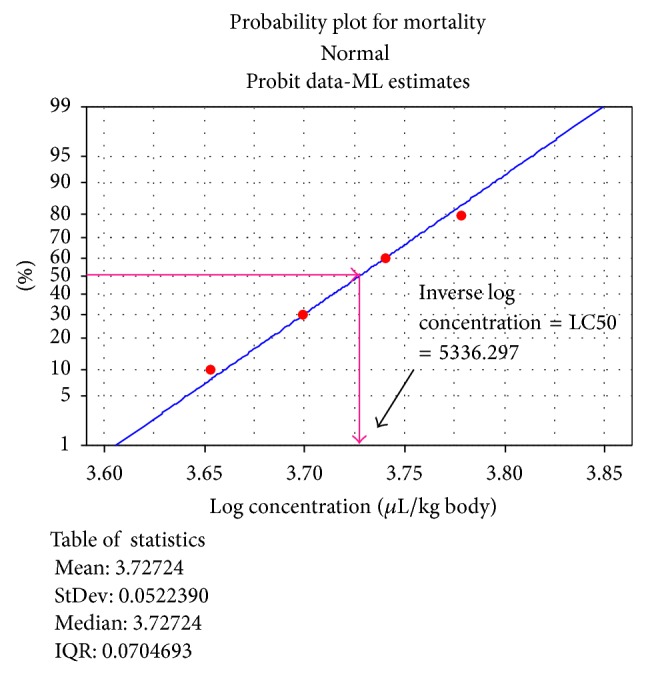
Probit transformed responses of mice treated with different concentrations of* T. ammi* essential oils after oral administration.

As it can be observed from Figure 2 that there was no mortality at the log concentration of 3,000 through 4,000 *μ*L/kg while there was one death at 4,500, two mortalities at a dose of 5,000, four mortalities at 5,500, and seven mortalities at 6,000 and 10 mortalities from 7,000. The LC50 was determined by drawing a vertical line on the *X*-axis from the point of the straight line the 50% mortality taken (Figure 2) and by calculating the inverse log of the value found on *X*-axis. The LC50 of the essential oils was, thus, 5336.297 *μ*L/kg body weight.

## 4. Discussion

In this millennium, infection from* Aspergillus* species becomes the major public health problem of modern mycology [24]. They have a capability to cause directly infection and indirectly mycotoxicosis especially upon the consumption of food contaminated with* Aspergillus* species. Many chemical preservatives have been used for the control of* Aspergillus* food contamination [25]. The widespread use of chemical preservative has significant drawbacks including increased cost, handling hazards, concern about pesticide residues on food, and threat to human health and environment [10]. Public awareness of these risks has increased interest in finding safer alternatives natural products to replace currently used synthetic chemical preservatives to control* Aspergillus* food contamination. One such alternative is the use of essential oils with antifungal and antiaflatoxigenic activities, since they tend to have low mammalian toxicity, less environmental effects, and wide public acceptance [17].* C. martinii, Foeniculum vulgare, *and* T. ammi* are common economic food spices in Ethiopia and, thus, it is an advantage to develop safe botanical food preservative against toxigenic* Aspergillus* species that have strong affinity to colonize various food commodities due to its secretion of hydrolytic enzymes [18].

Our TLC analysis confirmed the presence of various components of essential oils which were characterized by the distance they travel in a particular TLC system and their appearance (color) after visualization of the spots. Essential oils are very complex natural mixtures which can contain about 20–60 components at quite different concentrations [26]. They are characterized by two or three major components at fairly high concentrations (20–70%) compared to other components present in trace amounts [26]. This chromatogram developed from essential oils with the distinctive spot Rf and color were due to the presence of major component of essential oils: (i) alcohols: geraniol in* C. martini*; (ii) phenols: thymol and carvacrol in* T. ammi*; (iii) aldehydes: anisaldchyde in* F. vulgare*; (iv) ketones: fenchone in* F. vulgare;* (v) esters: geranyl acetate in* C. martinii*; and (vi) phenylpropanoids: anethole in* F. vulgare* essential oils [19]. And in our GC analysis we confirmed the presence of thymol (51.5%) and carvacrol (28.5%) as major components. TLC and GC finger prints are the most common chromatographic techniques widely available for phytochemical analysis of plant essential oils. They are used in standardizing the constituents of essential oils that are classified as generally recognized as safe by FDA for their use as food additives in controlling food spoilage [17].

Remarkable sporicidal activities against toxigenic strains of organisms tested were recorded in our study. Hundred percent inhibitions of spore germination were recorded at 4 *μ*L/mL for essential oil of* F. vulgare* and at 2 *μ*L/mL for essential oils of* C. martinii *and* T. ammi*. Similarly previous studies reported mycosporicidal activity of essential oils from* C. mertinii* [27], antimicrobial activity of* T. ammi *essential oils [24, 28], and antifungal effects of* F. vulgare* essential oils [29]. The impacts of essential oil on sporulation may be due to denaturation of the enzymes responsible for spore germination or interference with the amino acid involved in germination [30]. The vapor action exerted by volatile constituents of this essential oil on surface mycelial development and/or the transduction of signals involved in the switch from vegetative to reproductive development could also be responsible for the spore germination inhibition activity [30, 31].

Essential oils had a clear dose-dependent antifungal activity on* A. flavus* at the concentration tested in our agar dilution assay to determine MICs. Our finding suggests that the increment of dose to 2, 4, and 8 *μ*L/mL have a significant effect on the inhibition of fungal in reduction of fungal growth while all tested doses have significant effect in inhibition of spore germination. Absolute inhibition of fungal growth were seen at a concentration of 2 *μ*L/mL, 4 *μ*L/mL, and 8 *μ*L/mL for the essential oils of* T. ammi, C. martinii, *and* F. vulgare*, respectively, and the concentrations were recorded as MICs. Volatile constituents of the essential oils, might create vapor action which could be responsible for the activities [26]. Previous study reported antifungal property of essential oils, certainly, differences in major and minor constituents of the oils that are responsible for their biological activity by geographical location and seasons of collection and the difference in test organisms used in the study could contribute to the difference in MIC of* C. martinii* [32],* F. vulgare *[33], and* T. ammi *[34].

All essential oils have better fungitoxic activity than synthetic preservative used in our study, which was in accordance with the finding of previous study [26]. The inferior activity could be due to nature of the chemicals since it was reported that sodium benzoate was highly active at pH 3.5, weak acid compounds are more lipophilic in their nondissociated form which enables them to cross the cell membrane that led to pH lowering of cytoplamic cell with rupture of certain metabolic reactions of the microorganism, permeabilization of the cytoplasmic membrane, and cell death. Other authors demonstrated that benzoic acid has membrane-perturing potentials. In addition, these acids induce loss of mitochondrial function, and one possibility that we entertained was that this could be the result of mitochondrial autophagy [35]. Moreover, essential oils are complex mixtures of numerous molecules, and one might wonder if their biological effects are the result of a synergism of all molecules or reflect only those of the main molecules present at the highest levels. In the literature, in most cases, only the main constituents of certain essential oils like thymol, carvacrol, carvone, geraniol were analyzed [26]. Thus, synergistic functions of the various molecules contained in an essential oil, in comparison to the action of one or two main components of the oil, seem questionable. However, it is possible that the activity of the main components is modulated by other minor molecules [26]. Moreover, it is more likely that several components of the essential oils play a role in cell penetration, lipophilic or hydrophilic attraction, and fixation on cell walls and membranes, and cellular distribution [26].

Essential oil of* T. ammi *has pronounced activity in reducing mycelial biomass. The effects are dose-dependent, as up on the increment of the concentration of the oils the mycelia dry weights of the tested* Aspergillus *species were recorded. The reduction in fungal mycelia biomass may be due to the presence of phenolic compounds in the essential oils [26]. In addition, lypophilic nature of the essential oils helps to cross cell membrane of the fungal cell interacting with the enzymes and proteins of the membrane, thus producing a flux of protons towards the cell exterior which induces changes in the cells, and ultimately leading to death [26]. Similar findings were reported by previous study that showed* T. ammi* oils have the ability to reduce mycelial dry weight [36]. At low dose, phenols affected enzyme activity, especially of those enzymes associated with energy production while at greater concentrations, caused protein denaturation [36].

Another crucial finding of this study was that essential oil of* T. ammi *has antiaflatoxicogenic activity. Aflatoxin can be produced by* Aspergillus *species but the fungus may no longer be present in the food, hence preservative used for the control of aflatoxin should act on both fungus and the mycotoxin they produce. Interestingly, the productions of aflatoxin were inhibited by* T. ammi *essential oil at concentrations lower than recorded for MIC, spore germination inhibition, and mycelial dry weight. Thus, the inhibition of aflatoxin production cannot be completely attributed to reduced fungal growth, but may be because of inhibition of carbohydrate catabolism in* Aspergillus *species by acting on some key enzymes, reducing its ability to produce aflatoxins. Similar finding was reported from previous study [37]. Reduction of mycelial growth and spore germination inhibition and aflatoxin production inhibition could be due to the presence of thymol and carvacrol (phenolic OH group) that form hydrogen bonds with target enzyme active site [38].

Results from safety limit of* T. ammi *essential on mice showed that it is unlikely to present acute toxicity, supporting the consumption of this plant seeds as spice in Ethiopian community. All preservatives used against food spoilage moulds should not be harmful for human beings upon consumption. The safety limit of the* T. ammi *essential oil was also determined through its oral administration (acute oral toxicity) on mice and its LC50 value was found to be 5336.297 *μ*L/kg body weight and classified using WHO recommended classification in the U group [39]. The high value of LC50 is a symbol of the nonmammalian toxicity of the* T. ammi *essential oil, and hence it may be recommended as a safe preservative of food, as 5,000.00 *μ*L of the test substance/kg body weight is the practical upper limit for the amount of test material that can be administered in one oral gavage dose to a rodent. Nowadays using essential oils as food additives is common throughout the world [17].

## 5. Conclusions

Result of current study indicated that essential oils of plants possess antifungal property against toxigenic strains of* Aspergillus *species. We found that essential oils of* C. martinii, F. vulgare,* and* T. ammi* have fungitoxic potential. Moreover, essential oils of* T. ammi* have antiaflatoxigenic potentials and no mammalian toxicity on mice. Therefore, essential oils of* T. ammi* could be recommended as best safe botanical food preservative as it has antifungal as well as antiaflatoxigenic activity, superiority over synthetic fungicides, and nonmammalian toxicity. In addition, this essential oil has practical applicability as fumigant of food commodities due to their aromatic volatility nature. Moreover, we can also minimize the residual effect of this plant in food commodities by drying food staffs using sun light before consumption. As a result of these finding and opportunities we suggest* T. ammi *essential oil as a potential source of safe botanical food preservative that inhibits* Aspergillus* spore germination, growth, and mycotoxin production inhibition. However, further studies should be conducted to explore large scale utilization and also exploring the efficacy of* T. ammi* essential oils using other toxigenic organism that contaminate food commodities.

## Figures and Tables

**Table 1:** TLC finger print of essential oils of aromatic plants.

Plant	Spot (bands) Rf value and corresponding colors							
	1st	2nd	3rd	4th	5th	6th	7th	8th
Thy	0.51 (violet re)	—	—	—	—	—	—	—

Ta	0.52 (violet red)	0.45 (brown)	0.35 (gray)	0.23 (gray)	—	—	—	—

Cm	0.96 (t violet)	0.78 (blue)	0.73 (gray)	0.65 (blue)	0.54 (violet)	0.49 (blue)	0.39 (blue)	0.3 (blue)

Fv	0.95 (blue violet)	0.88 (violet)	0.69 (gray)	0.56 (violet)	—	—	—	—

Thy: thymol standard; Cm: *Cymbopogon martinii*; Fv: *Foeniculum vulgare*; and Ta: *Trachyspermum ammi*.

**Table 2:** Chemical constituents of fruit essential oil of *Trachyspermum ammi*.

Constituents	Retention time (min)	Percentage composition (%)
Thymol	22.305	51.5122
Carvacrol	25.505	28.5048
Unidentified	41.259	0.3734
*α*-terpinolene	42.972	1.8346
Unidentified	43.546	0.0273
*γ*-terpinene	44.691	5.1631
Unidentified	45.047	0.3331
*ρ*-cymene	45.721	6.1766
Unidentified	50.211	0.0976
Unidentified	50.543	0.0230
*β*-pinene	54.103	5.0505
Unidentified	54.597	0.0273
Unidentified	55.978	0.6719
Unidentified	56.431	0.2045

**Table 3:** Percent of spore germination inhibition of toxigenic *Aspergillus flavus* by essential oils using fungal spore germination assay.

Concentration (*μ*L/mL)	*C. martinii *	*F. vulgare *	*T. ammi *
0.25	49.57 ± 0.02^c^	32.57 ± 1.20^d^	88.14 ± 0.34^c^
0.5	75.14 ± 0.01^b^	45.71 ± 1.93^c^	94.86 ± 0.34^b^
1	98.57 ± 0.00^a^	81.14 ± 1.50^b^	100.00 ± 0.00^a^
2	100.00 ± 0.00^a^	99.80 ± 0.00^a^	100.00 ± 0.00^a^
4	100.00 ± 0.00^a^	100.00 ± 0.00^a^	100.00 ± 0.00^a^

Spore percent inhibition values are expressed as mean ± SD. Mean value with different letters in the same column are significantly different (*P* < 0.05).

**Table 4:** Antifungal activity of essential oils against the toxigenic strain of *Aspergillus flavus* using agar dilution technique.

Concentration (*μ*L/mL)	*C. martinii *	*F. vulgare *	*T. ammi *
0.25	23.05 ± 0.47^a^	23.09 ± 0.42^a^	22.71 ± 0.45^a^
0.5	21.19 ± 0.66^a^	21.62 ± 0.46^a^	17.28 ± 0.34^b^
1	18.52 ± 0.49^b^	19.10 ± 0.38^b^	0.00 ± 0.00^c^
2	16.76 ± 0.57^b^	17.14 ± 0.66^b^	0.00 ± 0.00^c^
4	0.00 ± 0.00^c^	0.00 ± 0.00^b^	0.00 ± 0.00^c^
8	0.00 ± 0.00^c^	0.00 ± 0.00^c^	0.00 ± 0.00^c^

Values are expressed as mean ± SD. Mean value with different letters in the same column are significantly different (*P* < 0.05).

**Table 5:** Comparative antifungal activity of essential oils with sodium benzoate.

*Aspergillus* species	MICs (mg/mL)			
	Cm	Fv	Ta	Sb
AFST	4.00	8.00	1.00	>16.00
AF001	4.00	8.00	1.00	>16.00
AF006′	4.00	8.00	1.00	>16.00
AF009	4.00	8.00	1.00	>16.00
AF019	4.00	8.00	1.00	>16.00
AF027	4.00	8.00	1.00	>16.00
AF037	4.00	8.00	1.00	>16.00
ANST	2.00	8.00	1.00	16.00
AN002	2.00	8.00	1.00	16.00

AFST: *Aspergillus flavus* (ATCC 13697); AF: *Aspergillus flavus*; ANST: *Aspergillus niger* (ATCC 10535); AN: *Aspergillus niger*; Cm: *Cymbopogon martinii*; Fv: *Foeniculum vulgare*; Sb: Sodium Benzoate; and Ta: *Trachyspermum ammi*.

**Table 6:** Percentage of growth inhibition of *Aspergillus *species on the basis of dry weight after treatments with different concentrations of *Trachyspermum ammi* essential oils.

*Aspergillus* species	Percentage of growth inhibition			
	0.25 *μ*L/mL	0.5 *μ*L/mL	0.75 *μ*L/mL	1 *μ*L/mL
AFST	11.93 ± 0.00^a^	43.07 ± 0.34^b^	71.18 ± 0.01^c^	100.00 ± 0.00^d^
AF001	24.23 ± 1.20^a^	51.06 ± 1.34^b^	73.05 ± 1.50^c^	100.00 ± 0.00^d^
AF006′	30.65 ± 0.10^a^	42.69 ± 0.93^b^	70.15 ± 0.01^c^	100.00 ± 0.00^d^
AF009	22.11 ± 0.34^a^	64.09 ± 0.34^b^	80.92 ± 1.34^c^	100.00 ± 0.00^d^
AF019	30.71 ± 0.01^a^	65.26 ± 0.02^b^	81.30 ± 0.01^c^	100.00 ± 0.00^d^
AF027	28.87 ± 0.01^a^	65.89 ± 1.20^b^	81.83 ± 0.02^c^	100.00 ± 0.00^d^
AF037	21.53 ± 0.03^a^	58.20 ± 1.93^b^	72.07 ± 1.20^c^	100.00 ± 0.00^d^
ANST	29.34 ± 1.02^a^	72.62 ± 2.34^b^	91.85 ± 0.01^c^	100.00 ± 0.00^d^
AN002	26.37 ± 0.02^a^	75.66 ± 0.57^b^	92.48 ± 0.34^c^	100.00 ± 0.00^d^

AFST*: Aspergillus flavus *(ATCC 13697)*; *AF: *Aspergillus flavus; *ANST: *Aspergillus niger *(ATCC 10535)*; and *AN: *Aspergillus niger. *Values are expressed as mean ± SD. Mean value with different letters in the same raw are significantly different (*P* < 0.05).

**Table 7:** Antiaflatoxigenic activity of *Trachyspermum ammi* essential oils at a concentration of 0.00, 0.25, 0.5, 0.75, 1, and 2 *μ*L/mL against toxigenic *Aspergillus *species.

*Aspergillus *	Aflatoxin production (fluorescence under UV 366*λ*)					
species	C∗	0.25 *μ*L/mL	0.5 *μ*L/mL	0.75 *μ*L/mL	1 *μ*L/mL	2 *μ*L/mL
AFST	+	+	+	ND	ND	ND
AF001	+	+	+	ND	ND	ND
AF006′	+	+	+	ND	ND	ND
AF009	+	+	+	ND	ND	ND
AF019	+	+	+	ND	ND	ND
AF027	+	+	+	ND	ND	ND
AF037	+	+	+	ND	ND	ND

AFST*: Aspergillus flavus* (ATCC 13697)*; *AF: *Aspergillus flavus; *+: aflatoxin production detected, ND: aflatoxin production not detected; and C∗: Tween 20 (5%).
